# Development and content validity testing of a colonoscopy-specific patient-reported experience measure: the Patient Experience Colonoscopy Scale (PECS)

**DOI:** 10.1186/s41687-024-00710-2

**Published:** 2024-03-18

**Authors:** Annica Rosvall, Malin Axelsson, Ervin Toth, Christine Kumlien, Magdalena Annersten Gershater

**Affiliations:** 1 Department of Care Science, Faculty of Health and Society, Malmö University, Malmö, Sweden; 2 Department of Gastroenterology, Skåne University Hospital, Malmö, Lund University, Lund, Sweden; 3 Department of Cardio-Thoracic and Vascular Surgery, Skåne University Hospital, Malmö, Sweden

**Keywords:** Cognitive interviews, Colonoscopy, Content validity, Content validity index, Face validity, Instrument, Patient-reported experience measure, Patient experience, Quality improvement, Questionnaire

## Abstract

**Background:**

In endoscopic care, favourable patient experiences before, during and after a colonoscopy are essential for the patient’s willingness to repeat the procedure. To ensure that significant experiences are measured, patients should be involved in creating the measurement instruments. Thus, the aim of the present study was to develop a colonoscopy-specific PREM by (1) operationalising patient experiences before, during and after a colonoscopy procedure and (2) evaluating its content validity.

**Methods:**

The colonoscopy-specific PREM was developed in two stages: (1) operationalisation with item generation and (2) content validity testing. A previously developed conceptual model, based on a systematic literature review that illustrates patients’ (*n* = 245) experiences of undergoing a colonoscopy, formed the theoretical basis. To assess the degree to which the PREM reflected patients’ experiences before, during and after a colonoscopy procedure, content validity was tested—through face validity with healthcare professionals (*n* = 4) and cognitive interviews with patients (*n* = 14) having experienced a colonoscopy. Content validity index (CVI) was calculated to investigate the relevance of the items.

**Results:**

The Patient Experience Colonoscopy Scale (PECS) is a colonoscopy-specific PREM consisting of five different constructs: health motivation, discomfort, information, a caring relationship and understanding. Each construct was defined and generated into a pool of items (*n* = 77). After face-validity assessment with healthcare professionals, a draft 52-item version of the PECS was ready for content validity testing by the patients. During cognitive interviews the patients contributed valuable insights that led to rewording and removal of items. Results from the CVI suggest that the PECS and its content are relevant (I-CVI range 0.5–1, S-CVI/Ave = 0.86). The final PECS consists of 30 items representing a colonoscopy-specific PREM.

**Conclusion:**

The PECS is a new 30-item PREM instrument designed for adult elective colonoscopy patients after they have undergone the procedure. Each item in the PECS derives from a conceptual model based on a systematic literature review. Patients and healthcare professionals were involved in developing the PECS, which measures colonoscopy-specific patient experiences before, during and after the procedure. The content validity testing positively contributed to the development of the PECS. Psychometric properties need to be evaluated further.

## Background

Colonoscopy is a fundamental procedure for diagnosis and treatment of clinical disorders of the lower gastrointestinal tract as well as for colorectal cancer screening [1–4]. However, patients who undergo the procedure may experience anxiety and may find themselves in an exposed and/or awkward situation and be embarrassed during the colonoscopy [5–9]. These negative experiences can be alleviated if the patients’ individual needs are met by the healthcare professionals, as such care promotes positive experiences [8, 10]. Which in turn pave the way for patients’ acceptance of and willingness to repeat the procedure [11–14].

Positive patient experience is associated with good care quality [15–18], and it is thus important for the healthcare to give the highest quality of care as well as gaining knowledge about how the patients experienced the colonoscopy procedure [19]. The patient perspective can be incorporated into care evaluation by collecting patient-reported experience measures (PREMs) through clinically relevant questions that matter to the patients [20]. This strongly aligns with the European Society of Gastrointestinal Endoscopy (ESGE) quality improvement initiative which recommends that patient experiences should be self-reported and measured routinely [21]. The PREMs should assess how the patients have experienced the provided care but also, and most importantly, provide support in identifying areas in need of improvement [22]. However, healthcare often fails to collect the measures that are most meaningful to patients [23] and that capture the essence of patient involvement when developing adequate measures [24].

There is no standard approach to measuring colonoscopy-specific patient experiences [21] and existing measures rarely report patient involvement during the development process [25]. Lack of patient involvement may lead to the value of the PREMs for the patients being questioned [25], since they, though experts of their own experiences [20], have not been a part of the development process [26].

Adult patients’ experiences of undergoing a colonoscopy was reported, in a systematic review, as a conceptual model developed by synthesising data from thirteen qualitative studies [27]. The conceptual model’s five main concepts were compared with eight existing multidimensional colonoscopy-specific PREMs, and the result showed that none of the identified PREMs fully covered the conceptual model [27]. These findings support and strengthen the argument for patient involvement during the development process of a colonoscopy-specific PREM.

## Methods

The aim of the present study was to develop a colonoscopy-specific PREM by (1) operationalising patient experiences before, during and after a colonoscopy procedure and (2) evaluating its content validity.

This is a psychometric study with both a quantitative and a qualitative design where the instrument in question was developed and validated in a two-stage process: Stage 1—operationalisation of the conceptual model including item generation, and Stage 2—evaluation of its content validity. The purpose of a new colonoscopy-specific PREM is to assess the quality of the care provided before, during and after colonoscopies by identifying potential areas of improvement. The target population for the instrument is adult patients who have undergone an elective colonoscopy.

### Operationalisation and item generation

The process of instrument development was inspired by Wolfe and Smith [28]. A conceptual model, based on a systematic review describing adult patients’ experiences before, during and after a colonoscopy procedure, formed the theoretical basis for the instrument [27]. In this study, colonoscopy-specific patient experiences are presented in five different constructs—*Health motivation, Discomfort, Information, A caring relationship* and *Understanding*—which correspond to the conceptual model [27].

Colonoscopy-specific patient experiences are abstract by nature and cannot be directly observed as a measure [29]. Due to this, an operationalisation process was required to identify the intended meaning of each construct and transform them into empirical observations [28]. The process started with definitions of the constructs, followed by specifications of the different domains in each construct. Subsequently, observable aspects of the domains were identified as indicators aiming to reflect how the domains might be directly observed [28] and answered by questionnaire respondents (Table 1). This was done in an iterative process, where the research group, during recurrent workshops, discussed the indicators until consensus was reached that each of them could be derived back to the conceptual model [27] and that they were related to clinical practice as well as to the target population.

**Table 1:** An overview of the constructs regarding definitions, domains, and indicators

Constructs	Time periods	Definitions	Domains	Indicators
Health motivation	Overall	Absence of disease and an interest in gaining **knowledge about their bowel health** motivate the patients to endure the procedure despite fear of a **potential diagnosis. Knowledge seems to support** the decision to proceed and reduce anxiety as well as increasing confidence in the colonoscopy	To determine bowel health	1. Wish to find out 2. Has to be undergone 3. To stay in line 4. To make sure everything is OK
			Thoughts about potential diagnosis	5. Worries about having cancer 6. Wanted to find out in time for treatment
			Knowledge	7. Knowledge about colorectal cancer 8. Knowledge about colonoscopy and the possibility of detection of anomalies 9. Go through the procedure regularly due to colorectal cancer screening
Discomfort	Before	A time when the patients may experience burdensome **specified preparations to empty the colon.** Patients with pre-existing conditions are likely to worry that the specified preparations may **exacerbate their medical conditions**. The time prior to colonoscopy is also characterised by **logistical planning** regarding transportation and time away from other duties	Impact on chronic diseases	1. For individuals with diabetes, worries to do with having to stay away from food, blood sugar levels and unclarity regarding insulin 2. Medical chronical conditions that impede the ability to drink large volumes
			Dietary guidelines	3. Lack of information 4. Tiresome to follow dietary guidelines and the required food preparation
			Bowel preparation	5. Uncertainty about instructions 6. Burdensome and inconvenient to drink large amounts of fluid with bad taste 7. Tolerated the bowel preparation due to motivation 8. Challenging to finish the bowel preparation 9. Nausea and/or vomiting 10. Feeling cold 11. Impact on sleep due to toilet visits 12. Painful to go to the toilet due to sore bottom
			Logistics	13. Transportation seen as a burden 14. Support with transportation home due to intake of sedation
	During	A time when the situation may be experienced as **exposed and embarrassing.** Experiences of different degrees of **discomfort and/or pain** may occur but can be eased with **sedation and/or support from staff**	Exposed situation	15. Embarrassment and an awkward situation 16. Overwhelming experience and disturbed safety
			Discomfort/pain	17. Unpleasant sensory experience 18. Different degrees of pain and peaks of pain
			Sedation	19. Sedated vs. awake according to wishes and personal preferences 20. Good effect of sedation in managing discomfort
			Support	21. Guidance from healthcare professionals’, e.g., regarding relaxation and breathing 22. Aided by physical contact 23. Aided by change in position 24. Aided by pausing the intubation of colon 25. Difficulties to verbally express discomfort 26. Difficulties to verbally express pain
	After	A time when the patients may experience **physical exhaustion and a need to recover,** as well as **changed bowel habits** and bloating	Exhausted	27. Physical tiredness due to lack of sleep or sedative/pain medication
			Impact on bowel habits	28. Changed bowel habits 29. Bloated
Information	Before	**Facts and understanding** regarding preparations and the process of undergoing a colonoscopy	Preparedness	1. Lack of information regarding diet and/or bowel preparation 2. Lack of information regarding the colonoscopy 3. Clear vs. confusing information
			Information seeking Verbal confirmation	4. Gathering of additional information 5. Source criticism
			Topic of conversation	6. Inappropriate to talk about and discuss a colonoscopy procedure with others
	During	**Sharing of information** regarding the procedure between healthcare professionals and the patient as well as **understanding** of the given information	Sharing of information	7. Fascinating vs. disgusting to watch the monitor 8. Explaining the procedure
			Understanding information	9. Problems remembering given information 10. Lack of full comprehension due to sedation 11. Persistent effect of sedation 12. Unanswered questions due to lack of knowledge or dizziness when discharged
	After	Either a preliminary or a definitive **colonoscopy result** given by the healthcare professionals to the patient after the procedure	The result	13. Lacked feedback concerning bowel preparation 14. Frustration due to lack of information 15. Reassuring to get the result 16. Result after procedure/recovery 17. Relief to get the result 18. Grateful to get the result
A caring relationship	During	A colonoscopy-specific caring relationship is based on the healthcare professionals’ **positive attitude and courtesy** towards the patient. In addition to this, it is essential that patients have **confidence in the competence** of the healthcare professional. **Respectful interactions** are a prerequisite for the creation of a caring relationships	Behaviour	1. Reassurance 2. Calm and comfort 3. Humour/Verbal praise 4. Nice manners/Well treated 5. Positive and friendly atmosphere 6. Feeling of being respected, safe and cared for 7. Treated as an individual
			Competence	8. Trust in the healthcare professionals’ knowledge
			Relationship-building interactions	9. Healthcare professionals’ responsive to individual needs, easing anxiety 10. Treated as a partner 11. In control due to the possibility to stop the examination if needed
Understanding	Before	For some patients, a time characterised by **fear of potential complications**. Patients without **former experience** of undergoing a colonoscopy are unsure of what will happen during the procedure and sometimes brood about the upcoming event. Negative previous experiences increase anxiety and serve as a barrier to undergoing the procedure, while positive previous experiences often reduce uncertainty and worries	Worries	1. Fear of complications 2. Concerns about safety and exaggerated negative expectations beforehand 3. Not knowing what to expect regarding sedation, pain, bowel movement
			Former experiences	4. Knowledge and understanding of the procedure 5. Previous experience prepared for the future
	After	A time characterised by a re-evaluation of the patients’ expectations prior to the colonoscopy and of their actual experience of undergoing the procedure. The patients’ **willingness to repeat the procedure** is affected by their assessment of the experiences	Willingness to repeat	6. Expectations not matching reality 7. View of the procedure changed afterwards 8. To experience a colonoscopy was demystifying

**Bold** text represents the essence

Out of all indicators, a pool of items was generated. The items were expressed as statements where complex sentences and difficult wordings were avoided. The answers should indicate the respondents’ level of agreement, based on their experiences of undergoing a colonoscopy, on a bipolar ordinal Likert scale with four response categories, from *strongly agree* to *strongly disagree* [30]. Mostly, high levels of agreement indicate a favourable experience, whilst low levels of agreement suggest a less positive experience. However, some items are reversed, meaning that high levels of agreement suggest a less positive experience and vice versa.

### Content validity

To assess the degree to which the items complied with the patients’ experiences, content validity was performed in accordance with COnsensus-based Standards for the selection of health Measurement INstruments (COSMIN) criteria, i.e., relevance, comprehensiveness and comprehensibility [31]. The content validity was tested through face validity with healthcare professionals and cognitive interviews with patients. In addition, the content validity index was calculated among patients to investigate the relevance of the items [32].

#### Face validity

Healthcare professionals at one endoscopy unit at a university hospital in the southern part of Sweden, with more than three years’ experience of endoscopic care, were invited to participate in the study during May 2022. Four healthcare professionals (two registered nurses and two endoscopists with a range of experience in endoscopic care from 5 to 23 years) accepted and were individually interviewed by the first author (AR). Two interviews were performed face to face, one was conducted via a digital platform and the last one by telephone. All interviews took place in a quiet room and the healthcare professionals were undisturbed. They were asked about the items’ clinical relevance and their wording, and about response categories and if they believed that the items were an adequate reflection of how they perceived patients’ experiences before, during and after a colonoscopy [33]. Additionally, they were asked if any key aspects were missing. The healthcare professionals’ reflections were noted and discussed by the research group before a first draft of the instrument was created for the cognitive interviews.

#### Cognitive interviews

To encourage the patients to reveal their detailed thoughts of the items’ meaning, cognitive interviews were used [34]. The data collection took place between May and November 2022 and was conducted at one endoscopy unit at a university hospital in the southern part of Sweden. The sample was prospective since all patients who met the inclusion criteria during three specified data collection periods, were invited. Patients who fulfilled the inclusion criteria of being adult (>18 years), Swedish speaking, able to participate in an interview and scheduled for an elective colonoscopy, received an invitation letter 2–4 weeks prior to the procedure. Out of 42 invited patients, 14 accepted study participation, 14 declined and 14 were either cancelled or re-scheduled for a colonoscopy and therefore did not match the inclusion criteria. The patients who accepted study participation were contacted by the first author (AR) within a week after their procedure for scheduling an individual interview (Table 2).

**Table 2:** Characteristics of patient participants (*n* = 14)

Characteristics	
**Age**	
Range in years (median)	29–81 (67.5)
**Biological sex (n)**	
Women	8
Men	6
**Educational level (n)**	
Secondary school	7
Higher education	7
**Occupation (n)**	
Employee	6
Retired	8
**Indication for colonoscopy (n)**	
Suspected cancer (e.g., rectal bleeding)	5
Other clinical bowel symptoms (e.g., suspected inflammatory bowel disease)	3
Colorectal cancer screening	4
Other indications (e.g., bowel adhesions)	2
**Colonoscopy experience (n)**	
First-time colonoscopy	5
Previous experiences of colonoscopy	9
**Bowel preparation at home (n)**	
Low-volume (1 L) polyethylene glycol (PEG) plus ascorbate solution (ASC)	10
Extended laxative with high-volume 3 + 2 L PEG solutions	2
**Sedation (n)**	
Conscious sedation (Midazolam^1^ and Oxycodone^2^)	10
Deep sedation (Propofol^3^)	1
None	3

^1^Midazolam 2,5–5 mg to 10 of the conscious sedated patients

^2^Oxycodone 2,5–5 mg to 10 of the conscious sedated patients

^3^Propofol 190 mg to the deeper sedated patient

They could choose the location for the interview, resulting in interviews via telephone (*n* = 5), via a digital platform (*n* = 4), at Malmö University (*n* = 4) and in the home of one patient (*n* = 1). During the cognitive interviews all patients were undisturbed and had the instrument at hand. Before the interviews began, the patients were introduced to the think-aloud method and instructed to verbalise their thought process while reading each item [35]. Patients were encouraged to give their view of the items rather than relay their experience of the colonoscopy. Nonetheless, for the PREM’s comprehensiveness, it was emphasised that the patients should highlight if any key aspect from their own colonoscopy experience was missing. For clarity, the patients were coached during the interviews, by means of concurrent probing questions, to further explain their reasoning [34]. The COSMIN criteria for content validity, regarding relevance, comprehensiveness and comprehensibility, were used [31] as a guiding principle for an interview guide during the interviews. The patients underwent the cognitive interviews within 21 days after their colonoscopy and the interviews lasted approximately 46 minutes (range in time 17–89 minutes). All interviews were audio recorded, transcribed verbatim and read individually by all authors in the research group, followed by joint consensus discussions. Interviews were conducted in four subsequent rounds, and after each round, adjustments, such as rewording and revision of the items, were agreed on, based on identified problems. After three rounds of interviews, no new data regarding the items were added. However, to deepen the understanding of comprehensibility regarding the response options, four of the patients who were available took part in a second interview. After the last round, no new issues needed to be addressed.

#### Content validity index

To determine the content validity index (CVI), six of the included patients were asked to assess each item for relevance on a scale ranging from 1 = not relevant to 2 = somewhat relevant, 3 = quite relevant and 4 = highly relevant [32]. A content validity index of individual items (I-CVI) below 0.78 was considered less relevant [36]. During consensus discussions, I-CVI values < 0.78 were weighted against the theoretical basis in the conceptual model to decide possible item removal. In addition, the average proportion of items that achieved a rating of 3 or 4 was calculated as S-CVI/Ave, and a value above 0.9 was considered excellent [32].

## Results

The developed PREM instrument was named the Patient Experience Colonoscopy Scale (PECS) and consists of five constructs and 25 domains: *Health motivation* (*n* = 3), *Discomfort* (*n* = 10), *Information* (*n* = 6), *A caring relationship* (*n* = 3) and *Understanding* (*n* = 3), as shown in Table 1.

### Operationalisation and item generation

During operationalisation, the colonoscopy-specific indicators (*n* = 75) were generated into 77 items. As an example, the indicator ‘*Responsive to individual needs, easing anxiety*’ was generated into an item as follows: ‘*I felt that my need for sedation/pain medication was met at the Endoscopy Unit*’. During workshops, the research group processed all items. When similar items arose, they were either modified or merged and item reduction was thereby achieved. This was, for example, relevant when items about feeling safe were present multiple times. During this process, 22 items were removed due to conceptual ambiguity. Furthermore, all three items concerning the domain *Logistical planning* were removed after consensus discussions in the research group, since the logistical issues that patients might experience with, for instance, transportation back and forth to the hospital, were perceived as out of the healthcare control area. This resulted in a draft 52-item instrument being developed for use in content validity testing.

### Content validity

**Fig. 1:**
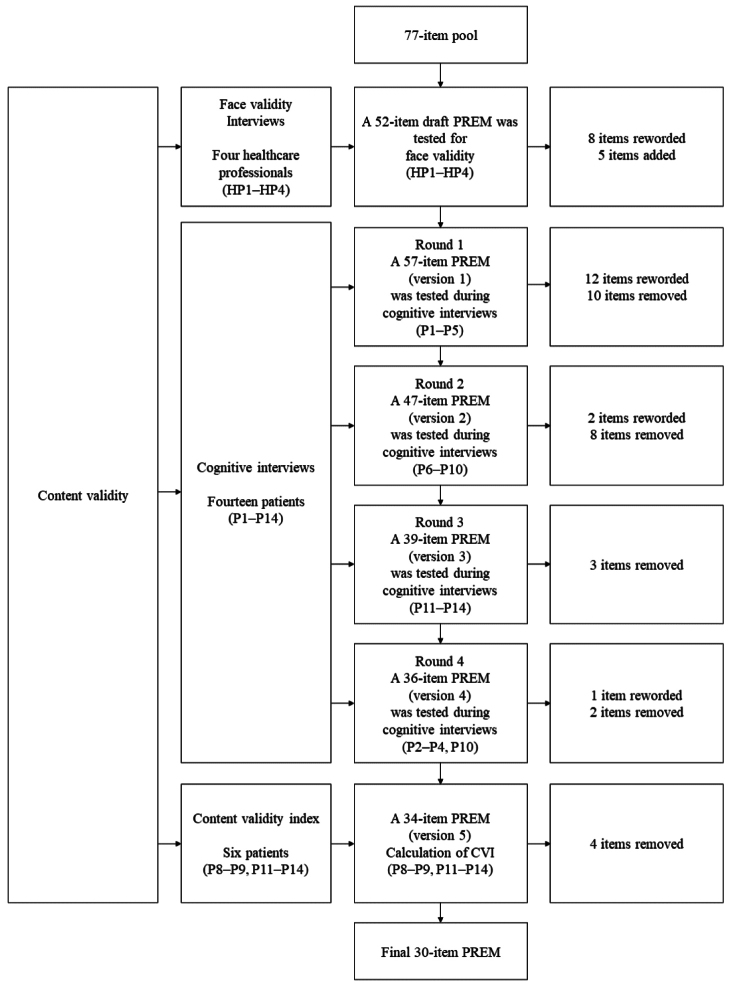
PREM development process, Stage 2—content validity

Healthcare professionals and patients were involved in the content validity development process (Fig. 1).

#### Face validity

During the interviews, the healthcare professionals stressed the importance of asking patients about their understanding of the given information, since adequate bowel preparation is clinically relevant and affects adenoma detection. They also stated that not all patients experience discomfort and/or pain during a colonoscopy and that sedated patients might suffer amnesia, which could cause uncertainties when answering items regarding information after the procedure. These insights led to the inclusion of several dichotomous screening items. In addition, feedback was given regarding negatively worded items, which led to positive rewording. The healthcare professionals highlighted problems with items being too close to one another, potentially measuring the same aspect of a domain, and therefore perhaps being difficult for patients to distinguish between when answering. The research group decided to keep the identified items regardless of this feedback, aiming to get additional feedback from the patients during the cognitive interviews. Furthermore, all healthcare professionals emphasised that the instrument consisted of too many items and that this might make it a challenge to use in clinical practice. Lastly, they thought that the items included seemed to reflect the constructs.

#### Cognitive interviews

The interviews with the patients were conducted in four rounds, and after each round, issues problematised by the patients were addressed, and revisions, such as rewording and removal of items, were made, prompted by the results (Table 3). Most items were considered relevant for the construct of interest. However, some items were considered less relevant and therefore removed.

**Table 3:** Examples of item revision based on cognitive interviews, rounds 1 to 4

57-item PREM version 1	Round 1 Cognitive interview findings	47-item PREM version 2	Round 2 Cognitive interview findings	39-item PREM version 3	Round 3 Cognitive interview findings	36-item PREM version 4	Round 4 Cognitive interview findings	34-item PREM version 5
*Health motivation – To determine bowel health*								
**Item 1. I did not feel hesitant to undergo the examination.**	**Relevance and comprehensibility** Participants found the item less relevant since several of them expressed that they didn’t have a choice: ‘*I’d rather avoid the colonoscopy, but that was not an option since I needed to undergo it*’ **Wording** The item was difficult to answer due to the negation: ‘*A bit tricky… you have to read properly*’. **Change** The item was reworded by removing the negation.	**I felt hesitant to undergo the examination.**	**Relevance and comprehensibility** All participants found the item easy to understand and considered it moderately relevant: ‘*I knew why I had to undergo a colonoscopy, so it was a foregone conclusion*’	**I felt hesitant to undergo the examination.**	**Relevance and comprehensibility** Participants described the item as redundant and as repeating a former question. One participant said, ‘*It is a relevant question, but what would you use that input for if I already answered that I understand why I have to undergo the examination*?’ **Change** No changes were made, since the research group considered the item important for the comprehensiveness of the construct Health motivation.	**I felt hesitant to undergo the examination.**	**Relevance and comprehensibility** Several participants found the item to be similar to another item, which was more positively worded: ‘It felt important for my health to undergo the examination’. **Change** After discussion within the research group, it was decided that the item should tentatively be exchanged for the more positively worded item, according to the participants’ suggestion after the calculation of content validity index.	**I felt hesitant to undergo the examination.**
*Discomfort – Impact on chronic diseases*								
**Item 2. I was not worried that the preparations for the colonoscopy would affect my state of health.**	**Relevance and comprehensibility** The item was considered less relevant. **Wording** The negation made it difficult to understand and answer. **Change** The item was reworded by removing the negation.	**I was worried that the preparations for the colonoscopy would affect my state of health.**	**Relevance and comprehensibility** The participants would rather answer a question about how worried they were concerning the bowel preparation, than a question about how it affected their state of health: ‘*I was worried about the laxative itself.. how it would affect me*’. **Change** The item was removed.					
*Discomfort – Impact on chronic diseases*								
**Item 3. I was not worried that stopping my regular medication would negatively affect my state of health.**	**Relevance and comprehensibility** Participants described the item as relevant. **Wording** The item was difficult to answer due to the negation. **Change** The negation was removed.	**I was worried that stopping my regular medication would negatively affect my state of health.**	**Relevance and comprehensibility** The item was described as relevant by the participants: ‘*I can understand that if you have medication, it’s a relevant question*’. **Wording** The item was difficult to answer if you didn’t have any medication. **Change** The answer alternative ‘I have no medication’ was added, as suggested by participants.	**I was worried that stopping my regular medication would negatively affect my state of health.**	**Wording** One participant stated, ‘*I have medication, but uh.. I didn’t have to stop taking it*. **Change** The answer alternative ‘I have no medication/I have medication but did not have to stop’ was added.	**I felt worried that stopping my regular medication would negatively affect my state of health.**	No problems reported.	**I was worried that stopping my regular medication would negatively affect my state of health.**
*Discomfort – Bowel preparation*								
**Item 4. My sleep was not affected by the bowel cleansing (laxation).**	**Relevance and comprehensibility** Participants considered the item less relevant. **Wording** The item was difficult to understand since it could be interpreted as referring to sleep being both positively and negatively affected. **Change** The wording was ‘not affected’ was replaced by ‘negatively affected’.	**My sleep was negatively affected by the laxative.**	**Relevance and comprehensibility** The item was hard to answer because participants’ sleep was disturbed by different causes: ‘*The anxiety affected my sleep. You might think that I needed to go to the toilet, but I didn’t, it was my dreams that woke me up*’. **Wording** The item was easy to understand.	**My sleep was negatively affected by the laxative.**	**Relevance and comprehensibility** Participants continued to try to interpret what the item referred to: ‘*The question probably refers to toilet visits at night..?’* **Change** The research group decided to keep the item due to the conceptual comprehensiveness, despite possible inconsistent answers.	**My sleep was negatively affected by the laxative.**	**Relevance and comprehensibility** Participants expressed that the item was highly relevant: ‘*The night before was difficult, I slept less than 4 hours due to toilet visits. In the morning I lacked energy’.* **Change** Due to ambiguities based on relevance, the item was retained pending the response from the content validity index calculation.	**My sleep was negatively affected by the laxative.**
*Information – Topic of conversation*								
**Item 5. It was easy to talk about having a colonoscopy with others outside of the healthcare system**	**Relevance and comprehensibility** Participants considered the item irrelevant. One participant, who had undergone the procedure several times, commented, ‘*Many people don’t know what a colonoscopy is, let alone how it is done*’. **Change** The cited reflection and the item’s lack of relevance led to removal of the item.							
*Understanding – Worries*								
**Item 6. Before the colonoscopy, I felt confident about how the procedure would be conducted.**	**Relevance and comprehensibility** The item was perceived as highly relevant, and the participants expressed the importance of information before the procedure.	**Before the colonoscopy, I felt confident about how the procedure would be conducted.**	**Relevance and comprehensibility** All participants found the item easy to understand and answer and perceived it as relevant. **Wording** One participant stated that ‘*you can’t be sure about anything*’ and this remark highlighted the need for exploring the word ‘confident’ in the next round of cognitive interviews.	**Before the colonoscopy, I felt confident about how the procedure would be conducted.**	**Wording** Participants were asked specifically about the wording and gave several suggestions, such as ‘well prepared’, ‘informed’ and ‘sure’. **Change** According to a suggestion from participants, ‘confident’ was changed to ‘informed’.	**Before the colonoscopy, I felt informed about how the procedure would be conducted.**	No problems reported.	**Before the colonoscopy, I felt informed about how the procedure would be conducted.**

The bold text is examples of different **items** during the development phase and the different versions of the PREM

It was also expressed that items that concerned the construct *Discomfort* and its domain *Dietary guidelines* were relevant, which was explained by the fact that several patients wished to give the staff quality improvement proposals regarding the information they had received. In addition, in the colonoscopy context, both conscious and deep sedation are common, although some patients choose to be awake during the procedure. This reality made it difficult for the patients to know what answer to choose regarding items that concerned the construct *Information*. Three out of five patients in the first round did not actually remember if they had been awake or asleep during the procedure, and this gave rise to the decision to keep items from the domain *Sharing of information* while the only item regarding the domain *Understanding information* was deleted. Nonetheless, none of the patients had difficulties recalling their overall experiences of the colonoscopy procedure.

All patients were asked at the end of the interview if they missed important aspects of a colonoscopy experience in the instrument and none of them thought that any key aspects were missing. With few exceptions, patients appeared to clearly understand the items as intended. However, simplifying rewordings were recommended by the patients, and the PREM introduction and the text were revised twice, according to those recommendations.

The patients were asked to share their thoughts about the response options. Most of them (*n* = 11) endorsed the response options and stressed that four alternatives were enough and that being ‘forced’ to ‘take a stand’ (positive/negative) was a good thing. However, two patients would have preferred dichotomous response options (yes/no), while one patient would have liked a response option in the middle (neutral) that would have enabled having no opinion. One patient suggested the use of numbers instead of words (1 to 4) for the response options, and another patient suggested simplifying the response options by changing *strongly agree* to just *agree*. Apart from these views, the response options were understood by all the patients as intended and therefore left without revision.

#### Content validity index

I-CVI values ranged from 0.33 to 1.00, and the S-CVI/Ave was 0.82. Nine items had an I-CVI below 0.78, and they were discussed in the research group, which resulted in keeping five of them due to their contribution to the conceptual comprehensiveness (Table 4). After the deletion of four items, the final PREM had I-CVI values that ranged from 0.50 to 1.00, and the S-CVI/Ave was 0.86.

**Table 4:** Overview of items whose I-CVI was < 0.78

Item	I-CVI	Consensus discussion	Action
Worries about stopping medication	0.5	Contributes to conceptual comprehensiveness	Kept
Important for health to undergo a colonoscopy	0.67	Contributes to conceptual comprehensiveness	Kept
Hesitancy to go through a colonoscopy	0.33	Negatively worded and similarities with another item	Deleted
Worries about getting a complication	0.67	Contributes to conceptual comprehensiveness	Kept
Negatively affected sleep	0.67	Inexplicitly worded and difficult to answer	Deleted
Information seeking	0.5	Beyond the control of the healthcare	Deleted
Interesting to watch the TV monitor	0.33	Irrelevant to several participants	Deleted
The colonoscopy went better than expected	0.67	Contributes to conceptual comprehensiveness	Kept
Would, based on previous experience/s, undergo a colonoscopy again	0.5	Contributes to conceptual comprehensiveness	Kept

After content validity testing, the PECS comprises 30 items forming five subscales which correspond to five constructs. The items are distributed over the time periods before, during and after a colonoscopy procedure (Table 5).

**Table 5:** Item distribution regarding constructs and time periods

Constructs		Time periods			Total
		Before	During	After	
Health motivation	3 items				3 items
Discomfort		7 items	5 items	2 items	14 items
Information		1 item	1 item	3 items	5 items
A caring relationship		–	3 items	–	3 items
Understanding		3 items	–	2 items	5 items
Total	3 items	11 items	9 items	7 items	30 items

## Discussion

In the current study, the PECS was developed through the operationalisation of patient experiences before, during and after a colonoscopy procedure. The PECS measures colonoscopy-specific patient experiences in an adult population after an elective procedure, and its content validity was tested according to COSMIN guidelines [31] where both patients and healthcare professionals were involved. The PECS is tentatively multidimensional and consists of 30 items, each of them derived from a conceptual model which describes and depicts how adult patients experience undergoing a colonoscopy [27].

The operationalisation of the colonoscopy-specific indicators resulted in a 52-item draft version of the PECS. This version had a clear theoretical basis, since the conceptual model from the systematic literature review included 13 qualitative research articles reporting how adult patients (*n* = 245) experienced undergoing a colonoscopy procedure [27]. This demonstrable connection to a conceptual model ensures that the scale is based on patients’ experiences and not on what healthcare professionals believe patients are experiencing, which is an important factor for capturing experiences that matter to the patients [20, 25]. A similar scale, the Newcastle ENDOPREM™, which aims to assess endoscopic patient experiences, apart from colorectal cancer screening [37], was developed using COSMIN guidelines [38] and cognitive interviews. However, for that scale the concept elicitation was based on interviews with only 10 patients who had undergone a colonoscopy [7], which may be considered a restricted theoretical approach [39]. In addition, the target population differs between the Newcastle ENDOPREM™ and the PECS, the latter being uniquely a colonoscopy-specific PREM intended for all adult patients that need to undergo the procedure.

Healthcare professionals’ concerns about the patients’ time to fill out a questionnaire, have been reported as a limitation for PREM usage [40]. The length of a questionnaire has an impact on response rates, where shorter is preferable [41]. In this study, the healthcare professionals gave valuable insights during face validity assessment when they highlighted the PECS as being too extensive with too many items for routine clinical use. Their comments prompted the balancing act of retaining a comprehensive instrument, where all constructs were represented, while developing a clinically useful instrument, where item reduction did not entail omitting any key aspects. This resulted in a 57-item version of the PECS that was due for further content validity testing with patients involved.

The cognitive interviews provided valuable insights into the respondents’ interpretation and comprehension of the items, and they confirmed that the statements reflected the constructs and domains as intended. However, during the four rounds of interviews some rewording was needed. For example, a statement that had to be reworded was the one regarding the impact on sleep (Table 1), an item that derived from patients describing their lack of sleep due to constant toilet visits caused by laxative during the night before the colonoscopy [9]. Not getting enough sleep during and after the bowel preparation causes exhaustion and tiredness [8, 13]. Nevertheless, the patients considered the first version of the item to be open to interpretation since sleep could be either positively or negatively affected, even though they assumed that the item most likely referred to sleep being affected in a negative way. This understanding resulted in a rewording of the item, where ‘*not affected*’ was changed to ‘*negatively affected*’. However, in the next two rounds, patients still expressed ambiguities, as sleep difficulties can be caused by different factors, such as bad dreams or, as reported by McEntire et al., by experienced anxiety prior to the colonoscopy due to fear of pain during the procedure or fear of the impending result of the examination [10]. After discussion within the research group, it was decided that the reworded item should be retained pending the response from the content validity index calculation. However, these insights emphasise the importance of cognitive interviews in uncovering the target population’s understanding of the items. Through the systematic capturing of the cognitive processes of the respondents, potential pitfalls that could compromise the content validity of the PECS were identified and rectified [42]. This approach not only contributed to the methodological rigour of the colonoscopy-specific PREM development but also ensured that the patients’ perspective was captured [43] and that the cognitive interviews worked as intended [42].

Individuals undergoing a colonoscopy are heterogeneous, making it challenging to generate items relevant to the entire target population. The result showed that nine items had unsatisfactory I-CVI (<0.78), and consequently four of those items were removed. An example of an item with unsatisfactory I-CVI was the statement regarding interest in watching the TV monitor during the colonoscopy (Table 4), where some patients found the item highly relevant while others considered it irrelevant, due to being asleep or sedated, or simply did not wish to see their intestines. Regardless, previous results show that unsedated patients experienced less pain and anxiety if they received detailed information while they watched the TV monitor during their colonoscopy procedure [44]. It is, arguably, challenging to find varied and relevant items that suit the whole population [45], and the relevance of this specific item was discussed both by the patients and in the research group throughout the whole development process. The I-CVI was 0.33, a result that played a decisive role when the research group finally decided to remove the item. However, not all items below 0.78 were deleted, because of their contribution to the conceptual comprehensiveness. Accordingly, the decision to retain five of the items with I-CVI values below the suggested value, in turn resulted in a S-CVI/Ave value slightly lower (0.86) than the recommended 0.9 [32].

### Strengths and limitations

The present study has, through a thorough theoretical approach, enabled the development of a colonoscopy-specific PREM. This was made possible by the process of operationalisation, including the identification of indicators that laid the foundation for item generation. Nonetheless, patient experience is a multidimensional construct [15] and when trying to fit reality into a specific construct, there is a risk that the theoretical underpinnings do not correspond entirely to the real world. Consequently, usage of the COSMIN methodology for content validity [31] provided conditions for testing if the content of the PECS was ‘an adequate reflection of the construct to be measured’ [33]. While researchers could be considered to be experts on theoretical concepts and on the operationalisation process to generate items, patients who have undergone a colonoscopy are the true experts of their own experiences. When these patients are involved in cognitive interviews, they can evaluate how the theoretical operationalisation process corresponds to reality and also suggest solutions for potential difficulties and ambiguities [42]. Moreover, for the purpose of revising the colonoscopy-specific PREM, between the four rounds of cognitive interviews, the whole research group participated in analysing the data, which may be considered a strength due to the researchers’ varied clinical competence and research experiences. Furthermore, in this study, the recruited patients were diverse concerning indication, colonoscopy experiences, sedation and bowel preparation, which is a strength since a variety of different patients’ perspectives were considered. Apart from the increased possibility to identify problems, this variety of patients also enhances the transferability of the PECS, in that it can be used in different settings.

The current study was carried out with a relatively small sample, which is common in qualitative research; hence the intention of the study is to confirm how the target population understands the items and not to generalise the results [23]. Furthermore, in the quantitative part of this study, the cut-off values (I-CVI > 0.78 and S-CVI/Ave > 0.90) suggested by Lynn [36] and recommended by Polit and Beck [32], were used, in order to minimise the element of chance and calculate the real agreement among the six raters. Even so, having six raters rather than fewer allows for a more diverse range of perspectives to be considered, which may contribute to a more consistent and accurate relevance assessment [46]. Moreover, one purpose of cognitive interviewing is to verbalise the participants’ thought processes [42], which means that this method assumes that the target population is able to provide such verbal reports [47]. However, it has been highlighted that not all cognitive processes can be verbalized [48]. In its current form, the PECS is only applicable in a Swedish colonoscopy context, due to the language, and the scale needs to be further tested for internal consistency [49] to confirm whether the number of items is appropriate. In addition, more advanced psychometric approaches, e.g., the Rasch measurement theory [50], are needed to further evaluate the PECS. Lastly, validity is a complex concept which may be examined from different perspectives and future studies can advantageously be designed according to modern validity theory when examine the PECS further [51, 52].

## Conclusion

Patient experiences are essential for healthcare quality and useful in evaluating provided care and identifying potential areas of improvement. Colonoscopy-specific patients’ experiences can be captured by a 30-item PREM named the PECS, which may, through its solid theoretical underpinnings, be a valuable addition to the endoscopic care and to future quality improvement initiatives. Both patients who have undergone a colonoscopy procedure and healthcare professionals have been involved in the development of the PECS, and it seems to contain key aspects of importance and be understood by the target population as intended, as well as consisting of items relevant to the constructs being measured. However, its psychometric properties need to be evaluated further.

## Data Availability

The datasets generated and/or analysed during the current study are available from the corresponding author on reasonable request.
